# The Formation of MgS & MgO Monomers and Dimers from Magnesium, Oxygen, and Sulfur Hydrides

**DOI:** 10.3390/molecules30081650

**Published:** 2025-04-08

**Authors:** Kailey M. Bell, Ryan C. Fortenberry

**Affiliations:** Department of Chemistry and Biochemistry, University of Mississippi, University, MS 38677, USA; kbell1@go.olemiss.edu

**Keywords:** astrochemistry, interstellar molecules, minerals, interstellar dust, rocky bodies, coupled cluster theory, quantum chemistry

## Abstract

The reaction of SH + MgH is shown in this quantum chemical study to be an energetically downhill pathway leading to the astronomically known MgS molecule as well as H_2_. Hence, the formation of MgS in the gas phase is now shown to be a possible contributor to this diatomic molecule’s observed astronomical abundance. Similarly, MgO can form through a related process with OH + MgH, but the energy costs are higher, implying that MgH could be consumed in reactions with SH faster than those with OH. Hence, MgS may be more abundant than MgO as a result in line with current astronomical observations. Additionally, further additions of MgS/MgO can lead to the dimers of these molecules, indicating that the formation of nanoclusters is possible. These structures could be the building blocks for larger dust grains and mineral-based materials that populate protoplanetary disks and, ultimately, lead to rocky bodies.

## 1. Introduction

Asymptotic giant branch (AGB) stars are late-stage stars that have consumed a majority of the hydrogen in their cores and are actively burning helium and heavier elements, dispersing these into in their surrounding shells and circumstellar envelopes [1]. These stars play a critical role in the chemical development of galaxies through their ability to produce and expel elements such as oxygen, carbon, nitrogen, and sulfur into the interstellar medium (ISM). These elements allow for more complex chemistry, such as dust formation, to occur [2], and this dust formation can aid in the development of more complex planetary systems and rocky bodies. Rocky bodies in planetary systems are known to be composed largely of minerals comprised of oxygen with other elements including silicon, magnesium, iron, sulfur, and aluminum, among others. Hence, the origin of these minerals may lie in inorganic oxide nanoclusters, but the formation of such clusters has only now been explored as a chemical problem [3,4,5,6,7,8,9,10]. The physical conditions in which such clusters could form from hydrides will dictate how the chemistry can progress.

The outer edge of AGB circumstellar envelopes can remain at temperatures as low as 25 K [11], on par with regions of the ISM, implying that chemical reactions relatively close to stars and those in the diffuse ISM may have many shared requirements. Given that temperatures in the outer edges of AGB star circumstellar envelopes can be well below cyrogenic levels, the primary source of energy for any reaction involving atoms or molecular hydrides of these newly produced atoms must largely come from the energy of the reactants themselves rather than external energy sources. As such, the energy of any intermediates and transition states in astrochemical reaction pathways, including those creating inorganic oxides, must remain lower than that of the initial reactants to indicate feasible mechanisms of formation in these environments. Furthermore, this resulting barrierless association between molecules results in an excess of energy that must be dissipated, such as through a leaving group to carry away the energy by-products as kinetic energy [12]. Unfortunately, the addition of two molecules without a leaving group (radiative association) typically results in the breakdown of the product back into the reactants [13] and is not as promising of a chemical reaction pathway with respect to gas phase formation as a leaving group would provide. Given the temperatures of most astronomical regions and the slowness of radiative association compared to back dissociation, a minimal (if any) energy barrier is required of plausible mechanisms for the formation of inorganic oxide clusters leading to dust particles.

The other consideration beyond energetics is the abundance of the starting materials themselves. Atomic magnesium, for instance, has been shown to be fairly abundant in the ISM and also a significant constituent of interstellar dust grains [14]. Sulfur and water derivatives are also highly abundant in the ISM [15,16], suggesting the potential for extensive chemical interactions between these components. Additionally, various metal hydrides are known in circumstellar media [17,18], and the hydroxyl and mercapto (SH) radicals have also been observed in the ISM [19,20]. Furthermore and in support of such chemistry, MgS has recently been detected in space, specifically towards the galactic center molecular cloud, G + 0.693 − 0.027, but the formation of this molecule is currently unclear, especially if any gas phase processes will contribute any major abundance to its astronomical population [21]. Hence, molecules comprised of metals and oxygen (or its sulfur analogues) may be present in astronomical regions where they could influence the creation of rocky bodies.

Previous quantum chemical computations have shown that the reaction of magnesium hydride with water is able to produce HOMgH with a leaving group of hydrogen gas [4,22], a molecule that has been dubbed “the solvent of the Universe” [23]. The addition of MgH_2_ to water first forms an intermediate in H_2_Mg-OH_2_. This is a fairly unstable compound because of the net exothermicity of this reaction with no place to dissipate the excess energy. This molecule then undergoes a transition state and ejects two of the hydrogens as H_2_. Such a mechanism allows the excess energy to dissipate as kinetic energy and two products, HOMgH and H_2_, are formed [4,24]. This reaction demonstrates both net exothermicity and submerged reaction barriers, making it a favorable pathway for the conditions present in the ISM. Expanding on these ideas, it may also be possible to form the cyclic dimers, *c*-Mg_2_O_2_ and *c*-Mg_2_S_2_, in similar pathways. This work aims to further investigate quantum chemical predictions for the earliest stages of grain formation from simple but known hydrides and assess whether these pathways can lead to the formation of larger inorganic oxide/sulfide molecules.

## 2. Results and Discussion

### 2.1. Magnesium Sulfides

**Figure 1:**
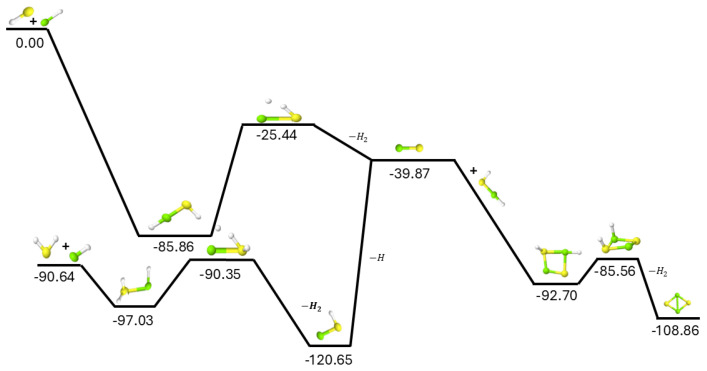
F12−TZ + ZPVE reaction pathway for MgH + SH and MgH + H_2_S. Relative energies are expressed in kcal mol^−1^ and scale vertically. Yellow represents S, green Mg, and white is H.

Since MgS has recently been detected in space and no viable gas phase mechanism exists for its provenance [21], the gas phase production of MgS, as shown in the upper-left of Figure 1, provides a novel pathway for the creation of this material from known astronomical molecules. The addition of the mercapto radical to magnesium monohydride (MgH) is able to barrierlessly form MgS through the downhill association into the HMgSH intermediate combined with hydrogen molecule production in the submerged TS and subsequent exothermic H_2_ ejection. This reaction is net exothermic, the first intermediate forms barrierlessly, the transition state energy is well-below the starting materials, and a leaving group in H_2_ can dissipate the exothermic energy kinetically. As such, the most viable pathway to form MgS in the gas phase may well be simply:

(1) $$\mathrm{SH}+\mathrm{MgH}\to \mathrm{MgS}+H_{2}.$$

Differently, and as shown in the bottom-left of Figure 1, the reaction of hydrogen sulfide (H_2_S) with MgH is able to form MgSH with an exceptionally small barrier in the TS. The relative value of 0.29 kcal mol^−1^ as compared to the starting materials is within the accuracy of the quantum chemical method, implying that this TS could actually be submerged. Regardless, if the barrier is 0.29 kcal mol^−1^ above the reactants, the external conditions required to bypass this barrier and produce MgSH would only need to reach a temperature of 30 K for 1% of the population of H_2_SMgH to overcome the TS barrier. However, the ejection of another hydrogen to form MgS from MgSH has an energy barrier of over 50 kcal mol^−1^, relative to the starting materials, or more than 80 kcal mol^−1^ relative to MgSH. This barrier for producing MgS from MgSH would be too great under any sensible astronomical conditions, as it would require a temperature of over 25,000 K for the barrier to be surmounted likely breaking the Mg-S bond first [3].

**Figure 2:**
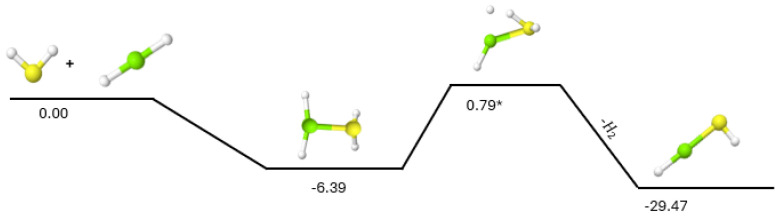
Reaction pathway for H_2_S + MgH_2_. Relative energies are expressed in kcal mol^−1^. The asterisk (*) indicates the barriered nature of the TS.

Similarly, the addition of H_2_S to magnesium dihydride (MgH_2_), as shown in Figure 2, is able to form HMgSH with another small energy barrier of 0.79 kcal mol^−1^ for the hydrogen ejection, equivalent to an external temperature of 388 K or requiring only about 90 K for 1% of the population to overcome this TS. Unfortunately, starting the reaction from H_2_S + MgH_2_ and producing HMgSH 29.47 kcal mol^−1^ below the reactants implies that the second H_2_ production TS is more than 30 kcal mol^−1^ above the starting materials. The pathway from HMgSH to MgS has already been shown in Figure 1 to require a total TS barrier for the second H_2_ production at roughly 60 kcal mol^−1^ relative to HMgSH. As such, the reaction of H_2_S + MgH_2_ will likely only produce HMgSH similar to what has been shown for H_2_S + SiH_2_ [24]. The lack of evidence for MgH_2_ in astronomical regions may imply that this reaction is likely not a notable contributor to the overall magnesium reaction network. If HMgSH can be produced, however, from either H_2_S + MgH_2_ or as the initial intermediate in SH + MgH, it actually can take part in some additional, useful chemistry.

The reaction of MgS with HMgSH is actually barrierless and creates a four-membered ring of alternating sulfur and magnesium atoms as shown on the right side of Figure 1. The net exothermicity of this is more than 50 kcal mol^−1^. The MgS adds directly onto the S and Mg atoms of the HMgSH, respectively, and the cyclic molecule forms with alternation of Mg and S atoms in the ring. As with the standard motif, construction of an H_2_ moiety produces a submerged TS with respect to MgS + HMgSH, and the hydrogen molecule departs the system translating the excess energy away from the stabilized, cyclic magnesium sulfide dimer. The TS for the departure of the H_2_ could not be fully located, but a scan produces an upper limit to the TS relative energy at −85.56 kcal mol^−1^ relative to the reactants which is still in line with the needed energies for this reaction pathway to progress. Regardless, if HMgSH can be formed and engage with MgS, nanostructures of magnesium sulfide may begin to arise requiring temperatures of less than 100 K to progress. The presence of HMgSH in regions where MgS has been observed would show the role that this molecule may play in further chemistry involving magnesium and sulfur.

The addition of Mg atoms to H_2_S, the atomic equivalence of MgH + SH, does not create a stable intermediate in MgSH_2_. While sulfur is well-known to create hemi-bonds [25] and magnesium is stable in similar bonding motifs as MgSH_2_ [26], the combination of magnesium atoms and hydrogen sulfide is not conducive for such bonding according to the present computations. Hence, Mg + H_2_S likely will not contribute to the formation of MgS, HMgSH, or the other minima on the potential energy surface.

### 2.2. Magnesium Oxides

**Figure 3:**
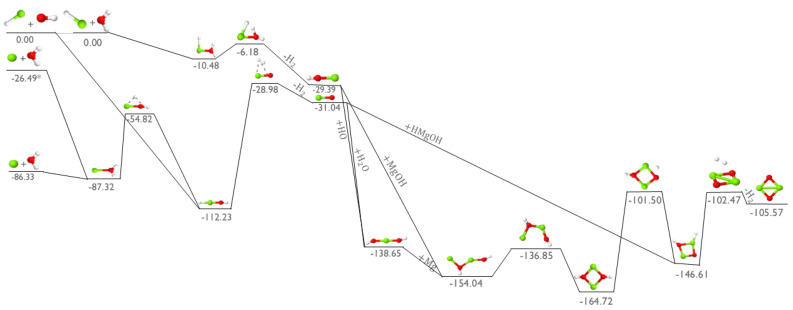
Reaction profile for the formation of MgO dimer. Relative energies are expressed in kcal mol^−1^. Red is oxygen, white is hydrogen, and green is magnesium. Reactants in their excited state are depicted with an asterisk (*).

In order to see if the analogous magnesium oxide (MgO) monomer can be produced in a similar fashion as the known MgS described above, Figure 3 depicts the addition of magnesium hydride (MgH) to OH in the upper-left. MgO is produced from OH + MgH in the middle of the figure with a barrierless association to HMgOH, an intermediate previously shown [4] to form from H_2_O + MgH_2_. However, the TS that leads to H_2_ is roughly 80 kcal mol^−1^ above the starting materials, 20 or so kcal mol^−1^ more than in the production of MgS. Additionally, the next exothermicity of OH + MgH → MgO + H_2_ is about 10 kcal mol^−1^ less than the equivalent reaction of SH + MgH. While MgO production is still exothermic, the larger exothermicity and smaller barriers for the formation of MgS could explain, in part, why MgS has been observed and why the upper limits for potential MgO abundance are less than those for its sulfur analogue.

The pathway for the initial addition of MgH to H_2_O (top pathway in Figure 3) leads to the relatively unstable HMgOH_2_ intermediate molecule which only lies 10.48 kcal mol^−1^ below the reactant starting materials. This molecule then requires overcoming an uphill, but submerged (relative to the reactants) TS to create and then eject an H_2_ molecule and produce MgOH with net exothermicity similar to the formation of its sulfur analogue, MgSH. MgOH likely cannot undergo a further O−H bond breakage and subsequent hydrogen atom ejection to form MgO due to the required large energy barrier computed to be on the order of +80 kcal mol^−1^. However, following the production of MgOH, another OH molecule can be added on to this molecule to produce the intermediate HOMgOH.

The two other reaction pathways initiated of the left-side of Figure 3 involve atomic magnesium reacting with water to create MgO as another possible means of producing this diatomic monomer. Unfortunately, the addition of ground-state, singlet, atomic magnesium to water is not as promising of a reaction to contribute to the formation of MgO. Notably, the H_2_O + Mg reaction faces a large energy barrier for the hydrogen isomerization to form HMgOH from MgOH_2_ and another, higher energy barrier for the H_2_ removal. This reaction would require large external energy input to undergo this process, likely requiring temperatures in the multiple thousands of Kelvin, and such conditions are not possible in the ISM. However, elemental magnesium in the excited triplet state may be more likely to interact with water to form MgO. The isomerization and H_2_ formation TS are submerged below the reactants, making it favorable in any temperature range as long as the 59.84 kcal mol^−1^ (as computed here; 21,850 cm^−1^ from experiment [27] which equates to 62.47 kcal mol^−1^, 458 nm, or 2.71 eV) excitation energy can be provided from some astronomical source.

Regardless of what the initial reactants are, if the MgO intermediate forms, it can receive a water molecule to form the same HOMgOH intermediate as OH addition to MgOH, shown in Figure 3. The relative energies of MgH + OH and MgH + H_2_O are equivalent for the creation of HOMgOH since these two pathways add the same three molecules, albeit in a different order, before intersecting at HOMgOH. Even so, when the MgO diatomic reacts with an additional water molecule over the OMg−OH_2_ scan coordinate, one O−H bond in the the H_2_O breaks with the hydrogen atom barrierlessly adding to the oxygen from the MgO to create HOMgOH. Once the Mg−O bond distance is less than 3.7 Å, the Mg−O bond is much stronger than the second O−H bond in water, and the net energy release from the OMgOH_2_ structure to HOMgOH is more than 80 kcal mol^−1^, making HOMgOH much more favorable. The HOMgOH molecule can exhibit different conformers with the cis−HOMgOH is slightly higher in energy than trans−HOMgOH. However, the barriers to conformational change and relative energies of the HOMgOH conformers are nearly zero with the differences computed here to be 0.1 kcal mol^−1^ or less.

Elemental, atomic, singlet magnesium can then be added to HOMgOH to form Mg_2_O_2_H_2_ as shown in the middle of Figure 3. The atom is selected to continue the reaction here since MgH is likely relatively scarce, and the atomic form will be more abundant. Alternatively, two MgOH molecules could add barrierlessly to form Mg_2_O_2_H_2_ directly. In either case from this minimum, a series of submerged isomerizations and an ultimate H_2_ creation and ejection ultimately form the Mg_2_O_2_ dimer. This exothermic pathway could then be the starting point for building larger inorganic oxide clusters that eventually aid in the formation of dust grains.

## 3. Computational Methods

The quantum chemical calculations employed in this work are performed using coupled cluster theory at the singles, doubles, and perturbative triples level [28] combined with the explicitly correlated F12b formalism [CCSD(T)-F12b] [29,30,31] and the associated triple-ζ correlation consistent basis set, cc-pVTZ-F12b [32,33]. This level of theory, referred to as F12−TZ, is used to optimize the geometries and compute the harmonic vibrational frequencies for all of the reactants, intermediates, and products (i.e., the minima) in this study via the Molpro 2022.3 quantum chemistry software [34]. Transition state (TS) optimizations are computed using Gaussian16 [35], employing density functional theory (DFT) at the B3LYP/aug-cc-pVTZ level of theory [36,37,38]. Once optimized and the single imaginary frequency determined, single-point energies are computed for the transition state geometries using F12−TZ again with Molpro 2022.3. The default parameters are used for all geometry optimizations and frequency computations. The zero-point vibrational energy (ZPVE) corrections, found using the harmonic frequency calculations, are included in the total energy calculations for the reactants, intermediates, products, and TSs included in the reaction pathways. The relative energies of each molecule in the reaction pathways are calculated with respect to the starting materials of each respective pathway in order to provide a reaction energy profile. This approach has been utilized previously and verified experimentally [39]. In the case that a TS geometry optimization is unsuccessful or direct dissociation is likely, a potential energy scan over a bond angle (with 5° increments) or bond distance (in 0.1 Å increments) is run in order to determine the behavior and/or upper limit of the relative energy.

## 4. Conclusions

The known interstellar molecule MgS can be formed through a straightforward reaction of known astronomical molecules SH + MgH to produce MgS + H_2_. This reaction pathway provides for a gas phase mechanism that has yet to be included in chemical reaction networks and may explain the formation of the observed MgS. Differently, neither reactions of hydrogen sulfide with MgH or that of H_2_S + MgH_2_ are energetically sound enough to create MgS as the second hydrogen loss, whether an an atom (in the former) or H_2_ molecule (in the latter) are exothermic compared to reactants. Even so, the production of HMgSH provides for the formation of *c*-Mg_2_S_2_ when this hydride reacts with MgS, also producing another H_2_ molecule in the process.

Similar chemistry is shown for the oxygen substitution where OH + MgH can lead to MgO + H_2_, but the energetics (and subsequent implied kinetics) of this reaction are not as favorable as with the sulfur analogues. HMgOH can also react with MgO to ultimately form *c*-Mg_2_O_2_+ H_2_ as with sulfur. However, the higher barriers will slow the reaction down. Previously computed rates of similar H_2_ elimination reactions on larger MgSiO_3_ and Mg_2_SiO_4_ clusters [10] have been predicted to be in the regime of 10^1^–10^3^ cm^3^ s^−1^, but the barrier to MgO + H_2_ from HMgOH is over 80 kcal mol^−1^, more than twice the previous. Hence, the rates here will be slower for MgO production from HMgOH, but the more comparable barrier for producing MgS should allow for similar rates. While OH is likely more abundant than SH in most astronomical sources, the lower barrier and faster kinetics for the MgH + SH reaction imply that in sources where both OH and SH could react with MgH, the MgH will be consumed first in reacting with SH giving rise to MgS in higher abundances than MgO.

Both reaction pathways imply that larger clusters of MgO or MgS could form, but the HMgOH and HMgSH intermediates would be required for additional monomer units to grow the cluster. The mechanisms explored here would require MgH_2_ as a starting material for stable HMgOH/HMgSH to form, and this molecule has yet to be observed, making its abundance likely low. As such, the monomers could be the effective termination or at least a major stopping point for magnesium oxide/sulfide chemistry in cold astronomical regions.

## Data Availability

All data for this work are contained within this manuscript or in the Supplementary Materials.

## Supplementary Materials

The supporting information can be downloaded at: https://www.mdpi.com/article/10.3390/molecules30081650/s1.

## Abbreviations

The following abbreviations are used in this manuscript:

AGB	Asymptotic Giant Branch
CCSD(T)	Coupled Cluster Singles, Doubles, and Perturbative Triples
TS	Transition State
MgS	Magnesium Sulfide
MgO	Magnesium Oxide
ZPVE	Zero-Point Vibrational Energy
kcal mol^−1^	kilocalorie mole^−1^
