# Supplementary material for: The Formation of MgS & MgO Monomers and Dimers from Magnesium, Oxygen, and Sulfur Hydrides
Source: Molecules. 2025 Apr 8;30(8):1650. doi: 10.3390/molecules30081650 (PMC12029866; doi:10.3390/molecules30081650)
Supplement: Supplementary file 1 [file molecules-30-01650-s001.zip › molecules-3549047-supplementary.pdf]

Supporting Information The Formation of MgS & MgO Monomers and Dimers from Magnesium, Oxygen, and Sulfur Hydrides

Kailey M. Bell and Ryan C. Fortenberry*

Department of Chemistry and Biochemistry, University of Mississippi, University, Mississippi, 38677, United States

E-mail: r410@olemiss.edu

S1 Cartesian Geometries in Å

All geometries are optimized with CCSD(T)-F12b/cc-pVTZ-F12 save for the TSs which are optimized with B3LYP/aug-cc-pVTZ.

OH

O 0.0000000000 0.0000000000 -0.0575076557

H 0.0000000000 0.0000000000 0.9128400371

OH_2

O 0.0000000000 -0.0657475499 0.0000004617

H 0.0000000000 0.5218202867 0.7574146023

H 0.0000000000 0.5218146015 -0.7574219310

MgH

Mg 0.0000000000 0.0000000000 -0.0693013561

H 0.0000000000 0.0000000000 1.6711009187

MgH_2

Mg 0.0000000000 0.0000000000 0.0000000000

H 0.0000000000 0.0000000000 1.7100959392

H 0.0000000000 0.0000000000 -1.7100959392

MgO

Mg 0.0000000000 0.0000000000 -0.6958822553

O 0.0000000000 0.0000000000 1.0571282808

HMgOH

Mg 0.0000441968 -0.0000424337 -0.6927593596

H -0.0005294541 -0.0014882585 -2.3926535118

O -0.0000370089 0.0006224582 1.0757476544

H 0.0000511702 -0.0073690212 2.0217973234

$HMgOH_2$

O -0.0261514074 -0.0088087988 -1.2213342234

Mg 0.0027342725 -0.0749077557 0.9070708726

H 0.0209794663 1.6823577600 0.9763591176

H 0.1128982495 0.8867019737 -1.5445885361

H 0.2153001455 -0.6229433994 -1.9177744939

MgOH

O 0.0000000000 -0.0028819602 -1.0209296988

Mg 0.0000000000 0.0006600120 0.7536545421

H 0.0000000000 0.0298311830 -1.9676875834

$MgOH_2$

O -0.0277502658 0.0002307329 -1.3519313938

Mg 0.0033555255 0.0000497070 1.0468437089

H 0.1804192636 0.7674406959 -1.8952739180

H 0.1791572531 -0.7723018143 -1.8881310500

TS-$HMgOH_2$

O 1.076406 -0.117181 -0.076275

H 1.874459 -0.331786 0.413354

Mg -0.914577 -0.115920 0.011556

H -0.181470 1.672726 0.035415

H 0.670688 0.987545 0.022760

TS-HMgOH

Mg 0.189451 -0.684853 0.000000

O 0.189451 1.067325 0.000000

H -1.687899 0.169401 -0.000000

H -2.101115 -0.489765 -0.000000

TS-$MgOH_2$

O -1.003392 -0.070169 0.015411

Mg 0.856383 -0.049893 -0.003230

H -1.958781 -0.171856 -0.079332

H -0.290676 1.331916 -0.005193

HOMgOH

Mg 0.000000000 -0.003318597 -0.001322726

O 0.000000000 0.053772902 3.367135112

O 0.000000000 -0.060410096 -3.369780564

H 0.000000000 -0.514334813 5.071605284

H 0.000000000 0.699712551 -4.997717450

HOMg_2OH

O 0.0000679632 -1.0050965922 -0.7692851761

Mg 0.0049143803 0.1465569635 0.7777537313

O -0.0091165302 0.1323145773 2.5682546943

H 0.0593321576 0.6357091187 3.3699561466

H 0.0074847015 -1.9657155011 -0.7868010284

Mg -0.0017288523 0.4831305577 -2.0690970055

TS-$Mg_2O_2H_2$ (ring closure)

Mg 1.054497 0.738816 -0.001206

Mg -1.653603 -0.801945 -0.000502

O -0.806947 1.039055 0.000636

H -1.366772 1.839023 0.004763

O 1.610687 -0.953492 0.001634

H 2.126127 -1.765978 -0.002421

Cyclic-$Mg_2O_2H_2$(adjacent H atoms)

Mg 0.000033463 0.098787842 -2.586127474

O 0.000026200 -2.600172157 -0.141295161

Mg -0.000076895 -0.373856246 2.469763875

O 0.000202210 2.658131186 0.630156378

H -0.001555990 4.465540677 0.678739586

H -0.001022332 1.247327030 -5.632674213

TS-$Mg_2O_2H_2$(H migration)

Mg 1.332943 0.228279 0.062977

Mg -1.365624 -0.288749 -0.017422

O -0.286196 1.359061 -0.026459

H -0.465606 2.312265 -0.076326

O 0.197904 -1.399170 0.090064

H 1.564117 -1.265745 -0.979178

Cyclic-$Mg_2O_2H_2$ (opposing H atoms)

O -0.0003878243 -1.2805600437 0.0000593401

Mg 0.0002438396 -0.0004392554 1.4767190220

O -0.0004611530 1.2815058169 0.0003970305

Mg 0.0002436939 -0.0002222191 -1.4770006318

H 0.0011955697 2.2361875658 -0.0000351018

H 0.0005244012 -2.2352496783 -0.0004184265

Mg_2O_2

Mg 0.0000000000 0.0000000000 1.2032522401

Mg 0.0000000000 0.0000000000 -1.2032522401

O 0.0000000000 1.4290633619 0.0000000000

O 0.0000000000 -1.4290633619 0.0000000000

SH

S 0.0000000000 0.0000000000 -0.0409156827

H 0.0000000000 0.0000000000 1.3014234839

SH_2

S 0.0000000000 -0.0548533475 0.0000001050

H 0.0000000000 0.8723741268 0.9647966420

H 0.0000000000 0.8723709177 -0.9647999811

HMgSH

Mg -0.0000366177 0.0015513598 -1.2789097168

S 0.0000114444 -0.0422129478 1.0281318927

H 0.0000102872 1.2986391362 1.1150684744

H 0.0005086766 0.0066382676 -2.9782823716

TS-HMgSH

S 0.000000000 0.000000000 0.000000000

H -1.916749208 2.102433697 0.115609665

Mg -5.166617049 -0.158056693 0.159502334

H -4.142880598 3.120780650 0.221783927

MgS

S 0.0000000000 0.0000000000 -0.9305866437

Mg 0.0000000000 0.0000000000 1.2275090639

H_2SMgH

S 0.0036037475 0.0562944056 1.2104958750

H 0.9552934268 -0.8844922123 1.2660117566

H -0.9590875374 -0.7759933916 1.6277600017

Mg -0.0739223124 -0.0074057820 -1.6490960734

H 1.6716966237 0.0484837879 -1.6310157282

TS-H_2SMgH

S 1.158049 -0.055931 -0.081707

H 1.273826 -0.082393 1.259805

H 0.143749 1.056629 -0.020529

Mg -1.576007 -0.139571 0.002698

H -1.034269 1.595515 0.035656

MgSH

S 0.0000000000 0.0420151185 -0.9855686419

H 0.0000000000 -1.3008036870 -1.0492934015

Mg 0.0000000000 -0.0014759363 1.3435488768

H_2SMgH_2

S 0.0564441958 -0.0002007194 -1.2075917935

Mg -0.0034455191 0.0002720487 1.5575088561

H -0.0212641386 1.7011256115 1.8432434100

H -0.0201541163 -1.6996379608 1.8517605796

H -0.8358887308 -0.9774208095 -1.4164390392

H -0.8349552300 0.9757574748 -1.4252005141

TS-H_2SMgH_2

S 1.198358 -0.090642 -0.082346

H 1.299942 -0.168112 1.258541

Mg -1.432139 -0.004287 -0.000380

H -2.680875 -1.147586 0.005460

H -0.870258 1.707339 0.056757

H 0.263132 1.110086 0.001346

$Mg_2S_2H_2$

S -0.0977669793 -0.0232290441 1.8961342602

Mg 0.0563034471 1.6618692395 -0.1633380913

H 0.3119571761 2.7569823048 -1.4887654912

Mg 0.1007530576 -1.4531397163 0.0071010594

S -0.0662254572 -0.2183633721 -1.8084505466

H 1.1170357879 -0.1057512777 2.4671979386

TS-$Mg_2S_2H_2$

Mg 0 0 0

S -0.1540704413 -1.685098401 2.059472498

Mg 0.04444961307 -3.115009184 0.1704391621

S -0.1225289086 -1.880232745 -1.645112579

H 1.060732416 -1.767620647 2.63053622

H 1.712902933 -0.1075656176 0.2752868496

Mg_2S_2

Mg 1.3220667946 0.0000000000 0.0000000000

Mg -1.3220667946 0.0000000000 0.0000000000

S 0.0000000000 0.0000000000 1.9026848849

S 0.0000000000 0.0000000000 -1.9026848849

S2 Harmonic Vibrational Frequencies (in cm^{-1})

All harmonic frequencies are computed with CCSD(T)-F12b/cc-pVTZ-F12 save for the TSs which are B3LYP/aug-cc-pVTZ.

OH

3740.70

OH_2

1650.86 3833.65 3943.58

MgH

1490.55

MgH_2

433.90 433.90 1599.41 1622.75

MgO

796.19

HMgOH

106.73 309.72 326.59 772.16 1635.31
4068.43

$HMgOH_2$

152.52 195.32 304.28 381.66 559.18

1453.15 1620.78 3797.11 3919.55

MgOH

75.40 758.93 4047.59

$MgOH_2$

55.17 103.75 241.38 1629.13 3758.14

3881.45

TS-$HMgOH_2$

1200.4801i 247.0769 500.6463

607.8558 882.5439 1287.7870

1518.6655 1843.6629 3851.8388

TS-HMgOH

Mg 0.189451 -0.684853 0.000000

O 0.189451 1.067325 0.000000

H -1.687899 0.169401 -0.000000

H -2.101115 -0.489765 -0.000000

TS-$MgOH_2$

O -1.003392 -0.070169 0.015411

Mg 0.856383 -0.049893 -0.003230

H -1.958781 -0.171856 -0.079332

H -0.290676 1.331916 -0.005193

HOMgOH

Mg 0.000000000 -0.003318597 -0.001322726

O 0.000000000 0.053772902 3.367135112

O 0.000000000 -0.060410096 -3.369780564

H 0.000000000 -0.514334813 5.071605284

H 0.000000000 0.699712551 -4.997717450

$HOMg_2OH$

105.00 109.03 168.74 183.15 257.37

315.31 427.65 490.78 667.35 784.31

3834.53 4021.15

TS-$Mg_2O_2H_2$ (ring closure)

Mg 1.054497 0.738816 -0.001206

Mg -1.653603 -0.801945 -0.000502

O -0.806947 1.039055 0.000636

H -1.366772 1.839023 0.004763

O 1.610687 -0.953492 0.001634

H 2.126127 -1.765978 -0.002421

Cyclic-$Mg_2O_2H_2$(adjacent H atoms)

186.36 250.10 329.04 342.16 367.05

393.65 560.49 622.82 646.31 735.06

1585.58 3938.75

TS-$Mg_2O_2H_2$(H migration)

Mg 1.332943 0.228279 0.062977

Mg -1.365624 -0.288749 -0.017422

O -0.286196 1.359061 -0.026459

H -0.465606 2.312265 -0.076326

O 0.197904 -1.399170 0.090064

H 1.564117 -1.265745 -0.979178

Cyclic-$Mg_2O_2H_2$ (opposing H atoms)

143.97 310.99 326.46 391.61 448.06

493.31 496.45 545.33 668.01 700.93

3935.36 3940.56

Mg_2O_2

284.56 447.07 571.15 605.67 662.12

670.98

SH

2700.22

SH_2

1213.64 2722.82 2738.00

HMgSH

287.86 311.91 432.50 517.67 1638.07

2683.89

TS-HMgSH

S 0.000000000 0.000000000 0.000000000

H -1.916749208 2.102433697 0.115609665

Mg -5.166617049 -0.158056693 0.159502334

H -4.142880598 3.120780650 0.221783927

MgS

526.75

H_2SMgH

124.03 148.66 202.18 257.80 461.29

1204.39 1482.84 2714.15 2730.47

TS-H_2SMgH

S 1.158049 -0.055931 -0.081707

H 1.273826 -0.082393 1.259805

H 0.143749 1.056629 -0.020529

Mg -1.576007 -0.139571 0.002698

H -1.034269 1.595515 0.035656

MgSH

410.72 477.22 2677.64

H_2SMgH_2

122.99 167.48 168.40 277.72 445.59

453.82 491.34 1206.05 1556.43 1570.99

2713.93 2730.35

TS-H_2SMgH_2

S 1.198358 -0.090642 -0.082346

H 1.299942 -0.168112 1.258541

Mg -1.432139 -0.004287 -0.000380

H -2.680875 -1.147586 0.005460

H -0.870258 1.707339 0.056757

H 0.263132 1.110086 0.001346

$Mg_2S_2H_2$

69.86 170.48 195.59 261.17 290.57

365.66 383.56 429.76 525.68 527.14

1499.39 2675.23

TS-$Mg_2S_2H_2$

Mg 0 0 0

S -0.1540704413 -1.685098401 2.059472498

Mg 0.04444961307 -3.115009184 0.1704391621

S -0.1225289086 -1.880232745 -1.645112579

H 1.060732416 -1.767620647 2.63053622

H 1.712902933 -0.1075656176 0.2752868496

Mg_2S_2

163.93 292.80 350.24 379.98 444.41

490.42
